# Surgery for Solitary Fibrous Tumors of the Pleura: A Review of the Available Evidence

**DOI:** 10.3390/cancers15164166

**Published:** 2023-08-18

**Authors:** Pietro Bertoglio, Giulia Querzoli, Peter Kestenholz, Marco Scarci, Marilina La Porta, Piergiorgio Solli, Fabrizio Minervini

**Affiliations:** 1 Division of Thoracic Surgery, IRCCS Azienda Ospedaliero Universitaria di Bologna, 40100 Bologna, Italy; marilina.laporta@studio.unibo.it (M.L.P.); piergiorgio.solli@gmail.com (P.S.); 2 Alma Mater Studiorum, University of Bologna, 40064 Bologna, Italy; 3 Pathology Unit, IRCCS Azienda Ospedaliero Universitaria di Bologna, 40100 Bologna, Italy; querzoligiulia@gmail.com; 4 Division of Thoracic Surgery, Cantonal Hospital of Lucerne, 6000 Lucerne, Switzerland; peter.kestenholz@luks.ch (P.K.); fabriziominervini@hotmail.com (F.M.); 5 Department of Thoracic Surgery, Imperial College Healthcare NHS Trust, London W2 1NY, UK; marco.scarci@mac.com

**Keywords:** pleural solitary fibrous tumors, surgery, minimally invasive surgery, overall survival

## Abstract

**Simple Summary:**

This is a review of the evidence on the use of surgery for solitary fibrous tumors of the pleura. Solitary fibrous tumors of the pleura are rare tumors that can arise in the chest from both visceral and parietal pleura. Surgery is the cornerstone of their treatment; as minimally invasive techniques, both thoracoscopy or robotics can be used according to the dimension, position, and infiltration of neighboring organs. A radical resection with free margins is the main target of surgery. Even if the long-term results are generally good, the risk of local or distant recurrence is always possible, in particular in cases of more aggressive histological types. In cases of local recurrence, surgery can be proposed if feasible.

**Abstract:**

Solitary fibrous tumors of the pleura (pSFT) are a relatively rare neoplasms that can arise from either visceral or parietal pleura and may have different aggressive biological behaviors. Surgery is well known to be the cornerstone of the treatment for pSFT. We reviewed the existing literature, focusing on the role of surgery in the management and treatment of pSFT. All English-written literature has been reviewed, focusing on those reporting on the perioperative management and postoperative outcomes. Surgery for pSFT is feasible and safe in all experiences reported in the literature, but surgical approaches and techniques may vary according to the tumor dimensions, localization, and surgeons’ skills. Long-term outcomes are good, with a 10-year overall survival rate of more than 70% in most of the reported experiences; on the other hand, recurrence may happen in up to 17% of cases, which occurs mainly in the first two years after surgery, but case reports suggest the need for a longer follow-up to assess the risk of late recurrence. Malignant histology and dimensions are the most recognized risk factors for recurrence. Recurrence might be operated on in select patients. Surgery is the treatment of choice in pSFT, but a radical resection and a careful postoperative follow-up should be carried out.

## 1. Introduction

Solitary fibrous tumors of the pleura (pSFT) are rare neoplasms arising from either visceral or parietal pleura, accounting for 4% of chest tumors with an incidence of 2.8 per 100,000 [1]. In the past decades, it was commonly known as “benign pleural mesothelioma” [2]. pSFT can occur at any age but is more frequently observed in middle-aged patients with a peak of incidence in the fourth and sixth decade (median in the fifth decade), with no gender predilection; moreover, some isolated cases in children and adolescents have been occasionally reported [3,4]. From a pathological point of view, according to the 2020 World Health Organization Classification, SFTs are classified as mesenchymal tumors of fibroblastic or myofibroblastic origin with intermediate biological behavior [2].

Although the most common site of onset is the pleura, similar mesenchymal tumors can arise anywhere in the body, such as the head and neck, thoracic wall, mediastinum, pericardium, retroperitoneum, and abdominal cavity; another described location includes the central nervous system, such as the meninges and spinal cord [5,6].

Biological behavior might vary from a benign tumor with an indolent course and a slow growth rate to a more aggressive tumor with the development of distant metastases [4]. Possible clinical manifestations are generally related to large masses and the most common symptoms are dyspnea, cough, pleuritic chest pain, shortness of breath, fever, and weight loss [3].

To date, the gold standard in the treatment of pSFT remains surgery, with the aim of a radical (R0) resection.

This review aims to analyze the role and the outcomes of surgery in the treatment of pSFT.

## 2. Methods

Given the rarity of pSFT, no prospective or randomized studies are available in the literature. For this reason, selected sources included case reports and observational and experimental studies of patients undergoing surgery for the diagnosis of pSFT.

### Search Strategy

We searched PubMed databases for RCTs, observational studies (prospective or retrospective), and case reports through to May 2023. PubMed was searched with the following keywords: “solitary fibrous tumor pleural” and “classification”, or “treatment”, or “surgery”, or “minimally invasive surgery”, or “outcome”, or “recurrence”, or “survival”, or “prognostic factors”. Only manuscripts with the full text in English were considered.

## 3. Histology and Molecular Features

From a histological point of view, SFT can be divided in two main phenotypes: hypercellular and hypocellular. pSFT are represented by a hypocellular phenotype, that is characterized by a thick collagenous background, often associated to hyalinized or collagen bands. Within the stroma, cells have a spindle shape, may be atypical, and they are arrayed haphazardly in a storiform configuration or in randomly oriented fascicle (“patternless pattern”) [2]. Areas with higher and lower cellularity usually alternate. On the other hand, the hypercellular type of SFTs are poor in collagen fibers and several solid nests are separated by capillaries. In this type of SFT, the cell shape is usually ovoid. A hemangiopericytic and perivascular pattern are the most characteristic histological patterns for SFT. Hemorrhage is common in cellular tumors, and necrosis may be present, particularly in malignant histotypes.

STAT6 is the most useful diagnostic marker, and it is expressed in 95% of cases. Other immunohistochemistry markers of SFT include positivity for CD34, Bcl2, CD99, and vimentin, while actin, desmin, S100 protein, or epithelial markers are negative. Nevertheless, there is a variability in the expression of the aforementioned markers.

From a molecular point of view, SFT typically show inversion of the long arm of chromosome 12 that brings to the fusion of the genes NAB2 and STAT6, which is typical of the macrofamily of SFT [7,8,9]. The NAB2 gene function is eventually involved in cellular differentiation and proliferation, while STAT6 acts as a transcriptional transactivator. The fusion of the two aforementioned genes is the driver of the tumorigenesis [8]. Interestingly, insulin-like growth factor-2 (IGF-2), which is implicated in the pathogenesis of Doege–Potter syndrome, one of the paraneoplastic syndromes that may be associated with pSFT, is dysregulated by the NAB2/STAT6 mutation [8].

Nonetheless, no molecular characteristics that are able to discriminate and stratify malignant and benign pSFTs are available so far. As a matter of fact, it has been suggested that some specific NAB2–STAT6 fusion could be related to a more aggressive biological behavior [10], but this finding has been questioned by other studies [11,12]

On the other hand, in up to 30% of all SFTs, a mutation in the promoter of the telomerase reverse transcriptase has been associated with worse prognostic outcomes and a more aggressive behavior [13,14,15], although the results of other studies are not consistent [16].

## 4. Classification

In the latest WHO classification (5th edition), the pSFT description encompasses various ICD-O codes (8815/0 solitary fibrous tumor, benign, 8815/1 solitary fibrous tumor NOS, 8815/3 solitary fibrous tumor, malignant). The effective prognostic role of conventional staging systems, such as the tumor, node, metastasis (TNM) staging system, is still not clear. The American Joint Committee on Cancer (AJCC)/Union for International Cancer Control (UICC) suggest to consider malignant pSFTs as soft tissue sarcomas, even though a clear and standardized definition of malignancies has not been released by these authorities [2].

For these reasons, many authors preferred to refer to a risk stratification model rather than anatomical staging. To better stratify clinical behavior and the subsequent risk of recurrence, different classification systems of pSFT have been proposed. All the classification systems are based on pathological or macroscopic features of the pSFT, and they aim to predict prognosis and the risk of recurrence. Due to the rarity of the tumors, the main bias of all the classification systems is to be based on the relatively small retrospective and often monocentric series.

Table 1 reports the characteristics off all of the proposed classification systems. All of them are based on histopathological characteristics: high cellularity with crowding and overlapping of nuclei, cellular pleomorphism, high mitotic index (with a general cut-off at four per high-power fields), necrosis or stromal/vascular invasion.

The first classification system was proposed by England [4] in 1989 and it was mainly based on histological features taking into account cellularity, mitosis, pleomorphism, necrosis, and presence of hemorrhage; dimension and other macroscopic feature were taken into account, but only the presence of one or more microscopic characteristics were related to the diagnosis of high risk (“malignant”) pSFT. In 2002, De Perrot and his coworkers [17] proposed to divide pSFT in four stages with a progressive correlation with worse outcomes according to macroscopic features and pathological characteristics. Together with the histologic features reported by England, the authors associate the presence of a pedunculated (lower risk) or sessile (higher risk) lesions.

The real prognostic impact of the latter classification has been discussed. Two retrospective series analyzed the impact of the De Perrot classification in their own institutional dataset of malignant pSFT. In both manuscripts, the authors failed to verify the prognostic relevance of this classification for overall survival [18,19]; on the other hand, Schirosi and colleagues [20] confirm the prognostic value of this classification.

More recently, in 2013, Tapias et al. [21] tried to standardize a classification and a score system to identify a reliable prediction of relapse that could help to address a long and appropriate follow-up only for patients with a high risk of recurrence. The scoring system was established, assigning one point to each of six tumor features. The scoring system was found to be predictive for tumor relapse when a score of ≥3 points was used as the cut-off, distinguishing low-risk patients from high-risk ones (the number of the patients for the validation of the score was too low to define an intermediate risk cohort). The same authors performed a follow-up study that, on one hand, showed a lower prognostic impact compared to the first study, but, on the other hand, it still had higher prognostic potential compared to [22].

In addition, Diebold et al. [23] conducted a retrospective analysis on 78 patients and reported a mean overall survival after surgery of 172 ± 13 months and a mean event-free survival of 165 (±15) months, with a median follow-up time of 36 months (range 1–216). Six patients (7.7%) had an adverse outcome, including pSFT relapse. Relying on these data, they proposed a scoring system expanding those from De Perrot [17] and Tapias [21] and introducing a new variable, namely the proliferation marker MIB-1.

**Table 1:** Table 1 reports all the elements included in different classification systems.

	England [4]	De Perrot [17]	Tapias [21]	Diebold [23]	Demicco [6]
**Histologic features for malignancy**					
Mitotic figures per 10 HPF	>4	>4	≥4	≥4	0
					1–3
					>4
Hypercellularity	Yes	Yes	Yes		
Pleomorphism	Yes	Yes			
Necrosis	Yes	Yes	Yes	Yes	Yes
Hemorrhage	Yes		Yes		
Stromal/vascular invasion		Yes			
**Anatomic feature for malignancy**					
Size	>10 cm		≥10 cm	≥10 cm	<5
					5–10
					10–15
					≥15
Peduncolated/sessile	Sessile	Sessile	Sessile		
Site	Parietal pleura		Parietal pleura		
**Biomarker risk factors**					
MIB-1 proliferation index				≥10%	
**Patients related risk factors**					
Age					≥55
**Criteria for malignancy**					
	One or more histologic criteria	One or more histologic criteria	Any three histologic and anatomic criteria	Two or more histologic, anatomic, or biomarker criteria	Low, moderate, or high risk according to the final score

Lastly, in 2017, Demicco [24] proposed another risk prediction model including patient age, tumor size, and mitotic activity; they then also introduced necrosis. This risk prediction model was not intended only for pSFT of the pleura and could be used also for extra-thoracic pSFT.

Several studies compared the different classification models; in their follow up study, as already mentioned, Tapias [22] showed a significantly better recurrence prediction of their model compared to that of England [4] and De Perrot [17]. Similarly, Ricciardi [18] found a significantly better prognostic role for the Tapias score compared to the De Perrot and Demicco, while Bellini [25] confirmed the prognostic impact of the England, Tapias, and Diebold scores, but not that by De Perrot.

## 5. Clinical Presentation

The vast majority of patients have no clear or specific symptoms at diagnosis; in some cases, intrathoracic masses, even of large dimensions, can be found incidentally at the time of the imaging test, which are carried out for other reasons with no symptoms reported. Regarding symptoms of dyspnea, cough or chest pain are the most typically reported [3,22,26,27]. Hemoptysis or pneumonitis has been described in case of large masses that cause atelectasis of the lung parenchyma [17,22].

Given the slow growth, pSFTs can reach impressive dimensions with poor or even no symptoms. The compression of large vessels or of the heart are the ultimate cause of signs that require further investigation and consequent diagnosis.

In rare cases, pSFT has been reported to be associated with paraneoplastic syndromes such as digital clubbing and hypertrophic pulmonary osteoarthropathy (HPO, Pierre–Marie–Bamberger syndrome); no clear mechanism bringing to the development of this syndrome has been found, which could be related to the secretion of cytokines and hyaluronic acid by the tumor and chronic hypoxia [28,29,30].

Furthermore, Doege–Potter syndrome has also been related to pSFT; this syndrome is characterized by a refractory hypoglycemia and involves less than 5% of all SFTs, even if it seems to be more common in large pleural or peritoneal lesions [31,32,33]. This syndrome is related to the secretion of insulin-like growth factor 2 (IGF-2) [34,35].

## 6. Surgical Management in the Treatment of pSFT

### 6.1. Preoperative Assessment

As discussed in the previous paragraphs, a diagnosis of pSFT is usually due to unexpected findings in chest radiological images or due to symptoms related to the dimension of the tumor.

Preoperative evaluation usually requires only a CT scan or, according to the surgeon’s preference, MRI. The pSFT does not have pathognomonic features and the characteristics are similar to those of other types of soft-tissue neoplasm. In more detail, pSFTs usually appear as homogeneous masses and are well defined; larger tumors may contain calcifications or cystic areas [36,37]. The neighboring organs are more frequently displaced rather than infiltrated [38,39]. Using MRI, pSFTs show low intensity T1 signaling and variable T2 signaling. The presence of a pedicle (which is reported in roughly 40% of cases) might result in a change in the location and shape of the pSFT [40,41]. In some cases, pleural effusion might be present [4,42].

In the case of small or well delineated lesions, MRI does not add further information compared to CT scanning, but it might be useful in cases of suspicious chest wall, vertebral, or diaphragmatic infiltration [27,38].

Beyond radiological tests, further exams, such as bronchoscopy, might be necessary in selected cases [30]. According to the extension of resection needed, pulmonary function tests might be necessary. Preoperative biopsy is not mandatory as the sensitivity has been reported to be low [43]; it might be performed in selected cases of unresectable advanced diseases or if a diagnostic doubt is present. Moreover, fine or core needle biopsies have a low sensitivity and malignant features are hard to identify with a small specimen [44].

Concurrently, ^18^FDG-PET scanning is not routinely required in the preoperative assessment of pSFTs [45]. Recently, a single-institutional study suggested [46] that ^18^FDG-PET scanning might have a role in stratifying the clinical behavior of pSFTs, identifying those with a more aggressive or malignant component. Yeom and colleagues [47] retrospectively reviewed preoperative CT and ^18^FDG-PET images of pathologically proven pSFTs and reported that morphological and metabolic features might help clinicians in the differential diagnosis; conversely, they could not predict the biological behavior of these neoplasms, but found a significantly higher standard uptake value (SUV) in patients with a malignant pSFT.

Lococo [48] reported the experimental use of ^68^Ga-Dotatoc in the preoperative workup of pSFTs, but only anecdotical further experiences using this technique has been reported so far [45], and, although results seem promising, no clear indications regarding its predictive and prognostic role are available to date.

In case of large masses, some authors suggest preoperative embolization of arterial vessels in order to reduce the risk of hemorrhage and minimize intraoperative blood loss [49,50]. It has been reported that up to 50% of pSFTs have a vascular pedicle that might arise form intercostal, internal mammary, or bronchial arteries [51,52]. In addition, abdominal feeding vessels from the abdominal aorta or celiac tripod have been reported [53,54,55]. In a case report, Song [56] reported the surgical ligation of the feeding vessel via an anterior thoracotomy followed by pSFT resection through a lateral thoracotomy. Nevertheless, no standardized indications for preoperative embolization are available and the decision is based on the multidisciplinary evaluation of each case.

### 6.2. Operative Approach

As in most oncological surgeries, the principles of pSFT surgery require a microscopically radical (R0) en bloc resection with a free safe margin [4,6,57]. Additional hilar or mediastinal lymphadenectomy is not recommended unless a clinical suspect is present [27,28,58]. As already mentioned, to date, the surgical approach represents the cornerstone of pSFT treatment and should be considered whenever possible. Yao and colleagues [59] analyzed data from the National Cancer DataBase (NCDB) of patients treated (either surgery or with other treatments) between 2004 and 2014 for pSFT; the authors found that the outcomes of those who received surgery were significantly better in terms of overall survival (64% versus 22% at 5 years).

The extent of surgical resection should vary according to the characteristics and features of pSFTs. When the tumor is pedunculated and it arises from the visceral pleura, a wedge resection often guarantees a radical resection with an adequate free margin; on the other hand, sessile tumors might require a wider parenchymal resection, which might range from a wedge resection to even a pneumonectomy in cases of larger or giant tumors [60]. Concurrently, in cases of pSFT arising from the parietal pleura, an extra-pleural preparation and resection is the gold standard with the addition of chest wall or spine resection if the tumor is invading chest wall structures. In case of doubt of radicality, a frozen section can be required during the surgical procedure [61]. In a relatively large series from a single center, Lahon and colleagues [27] reported 65% wedge resections, 10% lobectomies, and 3% pneumonectomy; interestingly, roughly 8% of surgeries required extended resections (chest wall, diaphragm, or vertebrae). On the other hand, more recently and based on the data of NCDB, Yao [59] found an almost equal rate of wedge resections and lobectomies (42% versus 45% respectively), but a higher rate of pneumonectomies (13%); the authors did not find a difference in OS when they compared lobectomies and wedge resections.

Different surgical approaches have been described, according not only to the period and the availability of technologies, but also, especially in recent times, to the dimension and the organ infiltration. Open surgery has been considered the gold standard approach; thoracotomy, sternotomy, or even hemi or complete clamshell, have been described in the literature [44,62,63]. Posterolateral was reported as the preferred access in different series [29,30,64] in cases of larger tumors.

In minimally invasive surgery, both VATS and RATS have been gradually introduced, given the known advantages in reducing postoperative pain and in-hospital stay. Minimally invasive surgery, even using a single port, can be offered in case of tumors of limited dimension, which allow safe and proper intraoperative manipulation, but the indication for the use of a minimally invasive technique is strictly related to the skills and knowledge of surgeons [44,65,66,67,68,69]. In a recent Chinese report [70], the authors found that patients treated with VATS had a significantly smaller diameter and a faster postoperative course compared to those treated with thoracotomy. Cardillo reported a conversion rate of 14.5% from VATS to open [71]; it must be noted that over time, the use of minimally invasive surgery has largely developed, and the conversion rate might have dramatically reduced. Nonetheless, patient safety and radical resection should be the priority for possible conversion from minimally invasive to open surgery.

Removal of the specimen in VATS and RATS must be performed with attention and by using a bag or by covering the utility incision with a soft tissue retractor in order to avoid possible contact metastases, which has been described by some authors [58]. In cases of large pSFTs, in order to avoid an enlargement of the minimally invasive surgical access and consequent injuries to the intercostal nerves, Hatooka [72] suggests use of a subxiphoid access.

### 6.3. Operative Management

Preoperative careful evaluation of imaging is mandatory for a safe surgical resection. As a matter of fact, intraoperative management strictly relies on the tumor dimensions and possible infiltration of the surrounding organs.

According to the tumor features, a 45° lateral decubitus position can be of extreme help in avoiding hemodynamic variations associated with decompressing the heart or great vessels [44]. A standard minimally invasive or open approach can be used in case of small or easily reachable tumors, while chest wall, diaphragm, or mediastinal structure invasion might require a dedicated surgical approach.

Intraoperative management should be customized in order to have the best exposure and to reach a R0 resection [66].

Intraoperative mortality in pSFT resection has been reported to range between 1.5% and 12% [44]; as already discussed, in case of large masses, the first step should be recognition and ligation of the feeding vessels in order to reduce bleeding and reduce intraoperative hemorrhage.

In cases of giant tumors (>20 cm) in the literature, an open approach is usually the treatment of choice. Association of different approaches, such as multi-level thoracotomies or thoracotomy and subcostal access, might be necessary to radically and safely resect giant pSFTs [56,73,74,75].

### 6.4. Postoperative Outcomes

The postoperative outcomes and the complication rate after surgery for pSFTs strictly depend on the extent of surgical resection and, quite obviously, on the dimension of the tumor. Bleeding and respiratory distress are the most reported complications. As reported before, particularly in cases of large tumors, preoperative embolization might help to prevent or reduce intraoperative bleeding [49,50,51,52].

In more detail, Lahon and coworkers [27] reported a single case of 30-day mortality and 5.7% of morbidity among 157 patients. Interestingly, almost 80% of patients that developed a postoperative complication had a malignant histotype; acute respiratory distress syndrome was the most common complication, while hemothorax was found in a single patient. The mean reported length of hospital stay was 11.5 ± 4 days. On the other hand, Tapias [21] reported a shorter length of stay (median 3 days, range 1–15), but they had a higher rate of minimally invasive surgery. Concurrently, Bellini [25] found a significant correlation between the presence of a giant tumor and intraoperative bleeding and a trend towards a longer in-hospital stay. In addition to this, in their manuscript focused on malignant pSFTs, Lococo and colleagues [26] highlight a 26% rate of postoperative complications (the most common were atrial fibrillation, bleeding, and pneumonia) and two cases of postoperative mortality; the first patient received an intrapericardial left pneumonectomy and the second one a lobectomy associated to a chest wall resection. Similar complications were also reported by a report from the Mayo Clinic, which additionally highlighted a 3.6% 30-day postoperative mortality rate [43].

### 6.5. Neoadjuvant and Adjuvant Treatments

Preoperative therapies have been seldom reported with no clear indications and, to date, are not recommended [17,76]. Out of 110 patients with SFT from several sites, Demicco [24] reported 9 cases who underwent preoperative chemo and/or radiotherapy. The possible influence of preoperative treatment on the long-term outcomes was not reported.

Similarly, the role of adjuvant therapies is still under debate as their real benefit has not been studied in large prospective studies, and results are often inconsistent [77,78,79,80].

Adjuvant radiotherapy might be suggested in patients with histological risk factors (e.g., higher mitotic count) or R1/R2 resections [24]. Suter and colleagues [81], in their own institutional series, reported a single case of adjuvant radiotherapy with excellent long-term outcomes. On the other hand, Cardillo reported a local recurrence [28] after chest wall resection and adjuvant radiotherapy (30 Gy) for a malignant pSFT. Adjuvant radiotherapy was also offered to 18 patients (20.9%) in a large retrospective study [79], which also involved extra-thoracic SFTs. The role of radiotherapy in the treatment of pSFTs has been also evaluated in a retrospective study based on 40 patients [82], that reported good long-term outcomes after definitive radiotherapy both in a curative (60 Gy) and in a palliative (39 Gy) setting (5 year OS 87.5% and 54.2%, respectively). It must be underlined that among the whole cohort, only nine patients had a pleural SFT (one in the definitive treatment setting and eight in the palliative setting). Radiotherapy has been anecdotally used for recurrence with promising results [80,83].

Similar to radiotherapy, the role of adjuvant chemotherapy has not been fully cleared and it is not standardized [17]. Chemotherapy has been described mainly for recurrence and the most common drugs that were reported are doxorubicin-based and gemcitabine-based [84]. More recently, research focused on the possible use of angiogenetic drugs, such as sunitinib, pazopanib, temozolomide, and bevacizumab [85,86,87,88]. In an in vitro study, Ghanim [77] reported the potential use of Trabectidin in association with Ponatinib. Despite the promising results, the data is currently not sufficient to establish new standards of care.

## 7. Long Term Results

### 7.1. Overall Survival

Overall survival after resection of pSFT is generally good. Table 2 reports main results of the largest and most recent study. Lahon and colleagues [27] reported a median OS of 14 years, a 5 year OS rate of 86%, and a 10 years OS of 77%; survival was significantly impaired in patients with a malignant pSFTs compared to those with a benign disease (5 year OS, 68% and 96%, respectively). Similar conclusions were shared by several other authors; Lococo and colleagues [26] reported an OS rate of 81% and 67% for benign and malignant pSFTs, respectively. Concurrently, in a more recent report with a median follow up of 91 months, Zhou and coworkers [63] did not report any death in the benign pSFT group and they found a 94.5 year OS, with a significant difference between benign and malignant disease. Similarly, Cardillo and his group [71] analyzed the outcomes of 110 patients who underwent surgery for pSFT and found a 10 year OS of 97.5. Lastly, in a small observational cohort study, Franzen and colleagues [89] found a pSFT-related mortality of 7.1%.

According to the majority of reported series, radicality of surgical excision (R0 resection) along with tumor size (larger or smaller than 10 cm), mitotic rate, presence of necrosis, and/or hemorrhage are well-recognized prognostic factors for pSFT [23].

### 7.2. Disease-Free Survival, Recurrence, and Recurrence Treatment

Table 3 reports features of recurrence. The recurrence rate for pSFT has been reported to be between 5% and 17%, but it grows up to an interval between 14 and 54% in case of malignant pSFTs [3,26,30,43,71]. Time to recurrence might be very long and it has been reported in case reports to be up to 17 years after surgery [90], but the majority occur in the first 2 years [76]. For this reason, both recurrence treatment and the correct follow-up period are not clear based on the current guidelines. The NCCN guidelines [91] include pSFT in the group of soft tissue sarcoma and recommend a very strict follow-up according to surgical results. Given the higher recurrence rate, more careful postoperative surveillance is required for malignant pSFTs with more strict controls and for a longer time compared to benign pSFTs [63]. The majority of the authors suggest a CT scan every 6 months for the first 2 years following surgical resection, then a yearly CT scan afterwards; PET scans may have a role in the follow-up if the first pSFT was PET positive [17,44].

A recent meta-analysis published in 202 based on 23 retrospective studies [92] found a 9% recurrence rate after surgery (even in case of R0 resections); the recurrence rate was significantly different according to the pathological features of the pSFT (benign and malignant: 3% and 22% recurrence rate, respectively); nevertheless, no significant difference was seen according to the site of origin of the tumor (visceral or parietal pleura). The impact of malignant histology on DFS has been confirmed by several authors [25,27,63,93,94]. On the other hand, Bellini and her colleagues [25] found a significantly worse DFS in patients who had a pSFT arising from parietal pleura. Moreover, in a multi-centric international study, Ghanim focused on circulating biomarkers as prognostic factors; the authors found that higher levels of fibrinogen were related with a significantly lower DFS, while the neutrophil–lymphocyte ratio (NLR) had no influence on DFS. Franzen [89] reported that the tumor diameter, the number of mitotic cells, and Ki67 expression as prognostic factors significantly influencing the DFS. Relapse might be systemic or, more frequently, local, close to resection margin. In case of local relapse, surgery can still be offered to patients if technically feasible and is usually considered the treatment of choice [17]. In their manuscript, Lahon and colleagues [27] reported re-surgery in 9 of the 10 cases with local recurrence; similarly, Lococo and his coworkers [26] reported an addition surgical resection in 4 patients out of 6 with local recurrence.

**Table 2:** Main results of recent studies reporting the outcomes of pSFTs treated with surgery. OS: Overall Survival; DFS: Disease-Free Survival; VATS: Video Assisted Thoracic Surgery; RATS: Robot Assisted Thoracic Surgery.

Authors and Year	Monocentric/Multi-centric	Number of Patients	Benign/Malignant	Type of Surgery	Open/Minimally Invasive	5 Year OS	10 Year OS	5 Year DFS	10 Year DFS	Follow-Up
Magdeilenat et al. 2002 [30]	Multicentric	60	38 benign 22 malignant	42 wedge resection 7 parietal pleura excision 11 extended resection	54 open (48 posterolateral, 5 anterolateral, 1 median sternotomy) 6 VATS	94%	94%	NA	NA	88 months (mean)
Harrison-Phipps et al. 2009 [43]	Monocentric	84	73 benign 11 malignant	62 wedge resection 2 segmentectomy 4 lobectomy 7 parietal pleura excision 9 extended resection	72 open (68 posterolateral thoracotomy, 3 median sternotomy, 1 transabdominal approach) 12 VATS	83%	NA	95%	NA	12 years (median)
Cardillo et al. 2009 [71]	Monocentric	110	95 benign 15 malignant	88 wedge resection 6 lobectomy 1 bilobectomy 2 pneumonectomy 12 parietal pleura excision 2 extended resection	41 open (40 thoracotomy, 1 median sternotomy) 69 VATS (16 conversion to open	NA	97.5%	NA	90.8%	12–222 months (range)
Lahon et al. 2012 [27]	Monocentric	157	90 benign 67 malignant	122 wedge resection 1 segmentectomy 15 lobectomy 3 bilobectomy 4 pneumonectomy 12 extended resection	142 open (139 posterolateral thoracotomy, 3 median sternotomy) 25 VATS (5 converted to open)	86%	77%	81%	74%	14 years (median)
Lococo et al. 2012 [26]	Multicentric	50	50 malignant	2 wedge resection 2 lobectomy 4 pneumonectomy 13 tumor excision 12 extended resection 3 explorative	NA	81.1%	66.9%	72.1%	60.5%	66.2 months (mean)
Tapias et al. 2013 [21]	Monocentric	59	NA	43 wedge resection 1 segmentectomy 4 lobectomy 1 bilobectomy 5 extended resection	45 open (41 thoracotomy, 2 median sternotomy, 2 thoracoabdominal access) 14 VATS	92.4%	83.4%	87.2	72.1	12.9 years (median)
Franzen et al. 2014 [89]	Monocentric	42	24 benign 18 malignant	75.6% wedge resection 17.1% lobectomy 7.3% pneumonectomy	22 open 20 VATS	NA	NA	84%	67%	39 months
Ghanim et. al. 2017 [94]	Multicentric	125	104 benign 21 malignant	NA	91 open 32 VATS 2 RATS	NA	NA	77%	67%	NA
Diebold et al. 2017 [23]	Multicentric	78	NA	82% wedge resection 12% lobectomy 6% pneumonectomy	39 open (thoracotomy) 39 VATS	NA	172 months (median OS)	NA	93%	NA
Tan et al. 2018 [70]	Monocentric	82	70 benign 12 malignant	39 wedge resection 7 lobectomy 1 pneumonectomy 35 extended resection	60 open (thoracotomy) 22 VATS	NA	76% (malignant)	NA	53% (malignant)	56 months (median)
Bellini et al. 2019 [25]	Multicentric	107	79 benign 28 malignant	16 tumor resection 79 wedge resection 12 extended resection	81 open (70 thoracotomy, 1 median sternotomy, 1 sternolaparotomy, 2 clamshell) 26 VATS	NA	NA	NA	81%	7 years (median)
Zhou et al. 2020 [63]	Monocentric	70	58 benign 12 malignant	30 wedge resection 6 lobectomy 2 pneumonectomy 29 parietal pleura excision 3 extended resection	43 open (37 thoracotomy, 6 median sternotomy) 27 VATS (3 conversion)	100% (benign)	100% (benign) For malignant patients: median OS 83 months	100% (benign)	100% (benign)	91 months (mean)

**Table 3:** Main results of recent studies reporting recurrence features after surgery. The results of local and distant recurrence could be higher than the total number in cases where patients experienced both local and distant recurrence. NA: Not Available.

Authors and Year	Number of Patients	Number of Recurrence (Total)	Number of Recurrence (Benign)	Number of Recurrence (Malignant)	Number of Local Recurrence	Number of Distant Recurrence	Treatment of Recurrence
Magdeleinat et al. 2002 [30]	60	3	0	3	2	1	2 Surgery 1 Unknown
Cardillo et al. 2009 [71]	110	8	4	4	NA	NA	3 Surgery 4 Surgery and radiotherapy 1 Surgery and chemotherapy
Lahon et al. 2012 [27]	156	15	1.3%	19%	10	5	9 Surgery 1 Chemoradiotherapy 3 Radiotherapy 1 Chemotherapy 1 Unknown
Lococo et al. 2012 [26]	50	15	-	15	6	9	4 Surgery 11 Chemotherapy
Tapias et al. 2013 [21]	59	8	NA	NA	8	0	8 Surgery
Franzen et al. 2014 [89]	84	8	2	6	3	5	4 Surgery 4 Unknown
Ghanim et al. 2017 [94]	125	14	6	8	11	3	9 Surgery 5 Chemotherapy or radiotherapy
Tan et al. 2018 [70]	82	4	0	4	4	0	3 Surgery 1 Conservative treatment
Bellini et al. 2019 [25]	107	12	5	7	7	7	4 Surgery 4 Surgery and radiotherapy 1 Surgery and chemoradiotherapy 1 Chemotherapy 2 Unknown

## 8. Conclusions

Pleural SFT a relatively rare type of tumor that arises from the pleura. Thoracic surgeons should consider pSFT in their differential diagnosis in case of masses with variable dimensions that have high contact with the pleura. Despite the absence of prospective studies and standardized guidelines for their treatment, surgery should be always considered and should be planned and carried out with the aim of radical surgery, which might require extended resections. Postoperative surveillance is also of paramount importance as recurrence is possible, especially in tumors with more aggressive features, and surgery should be considered also in cases of local recurrence, if technically feasible.

Prospective studies and a more standardized classification are needed in the future to better stratify the treatment, risk of recurrence, and follow-up of these patients.

## Data Availability

The data presented in this study are available in this article.
